# The Reversal Effects of 3-Bromopyruvate on Multidrug Resistance *In Vitro* and *In Vivo* Derived from Human Breast MCF-7/ADR Cells

**DOI:** 10.1371/journal.pone.0112132

**Published:** 2014-11-05

**Authors:** Long Wu, Jun Xu, Weiqi Yuan, Baojian Wu, Hao Wang, Guangquan Liu, Xiaoxiong Wang, Jun Du, Shaohui Cai

**Affiliations:** 1 Department of Clinical Pharmacology, College of Pharmacy, Jinan University, Guangzhou 510632, P. R. China; 2 Division of Pharmaceutics, College of Pharmacy, Jinan University, Guangzhou 510632, P. R. China; 3 School of Pharmaceutical Sciences, Sun Yat-sen University, Guang Zhou 510275, P. R. China; Wayne State University School of Medicine, United States of America

## Abstract

**Purpose:**

P-glycoprotein mediated efflux is one of the main mechanisms for multidrug resistance in cancers, and 3-Bromopyruvate acts as a promising multidrug resistance reversal compound in our study. To test the ability of 3-Bromopyruvate to overcome P-glycoprotein-mediated multidrug resistance and to explore its mechanisms of multidrug resistance reversal in MCF-7/ADR cells, we evaluate the *in vitro* and *in vivo* modulatory activity of this compound.

**Methods:**

The *in vitro* and *in vivo* activity was determined using the MTT assay and human breast cancer xenograft models. The gene and protein expression of P-glycoprotein were determined using real-time polymerase chain reaction and the Western blotting technique, respectively. ABCB-1 bioactivity was tested by fluorescence microscopy, multi-mode microplate reader, and flow cytometry. The intracellular levels of ATP, HK-II, and ATPase activity were based on an assay kit according to the manufacturer’s instructions.

**Results:**

3-Bromopyruvate treatment led to marked decreases in the IC_50_ values of selected chemotherapeutic drugs [e.g., doxorubicin (283 folds), paclitaxel (85 folds), daunorubicin (201 folds), and epirubicin (171 folds)] in MCF-7/ADR cells. 3-Bromopyruvate was found also to potentiate significantly the antitumor activity of epirubicin against MCF-7/ADR xenografts. The intracellular level of ATP decreased 44%, 46% in the presence of 12.5.25 µM 3-Bromopyruvate, whereas the accumulation of rhodamine 123 and epirubicin (two typical P-glycoprotein substrates) in cells was significantly increased. Furthermore, we found that the mRNA and the total protein level of P-glycoprotein were slightly altered by 3-Bromopyruvate. Moreover, the ATPase activity was significantly inhibited when 3-Bromopyruvate was applied.

**Conclusion:**

We demonstrated that 3-Bromopyruvate can reverse P-glycoprotein-mediated efflux in MCF-7/ADR cells. Multidrug resistance reversal by 3-Bromopyruvate occurred through at least three approaches, namely, a decrease in the intracellular level of ATP and HK-II bioactivity, the inhibition of ATPase activity, and the slight decrease in P-glycoprotein expression in MCF-7/ADR cells.

## Introduction

Breast cancer is one of the most critical threats to women, and its incidence is increasing year by year [1]. Chemotherapy and endocrine therapy are still predominantly used for the treatment of breast cancer. While advancements in breast cancer treatment and prevention have emerged over the last decade, multidrug resistance (MDR) has been a main cause of breast cancer chemotherapy failure [2]. The mechanisms underlying MDR are rather complex, and among them, transporter-mediated efflux is a major one that has received enormous attention [3], [4]. The efflux transporters, including P-glycoprotein (ABCB-1/P-gp)[5], multidrug resistance proteins (MRPs) [6], and breast cancer resistance protein (BCRP) [7] are over-expressed in many cancer cells, limiting the entry of the drug into the inside of cells and conferring the resistance of cells to the drugs [4]. P-gp belongs to the ABC transporter family (ABC) with seventeen trans-membrane helices and two ATP-binding domains [2]. The physiological expression of P-gp protein has been found in liver, intestine, and blood-brain barrier. Many anticancer drugs (e.g., doxorubicin, paclitaxel, daunorubicin, and epirubicin) are substrates of P-gp [8]. However, it is still difficult to predict P-gp activity toward a new compound, although many structure-activity relationships have been established.

**Figure 1:**
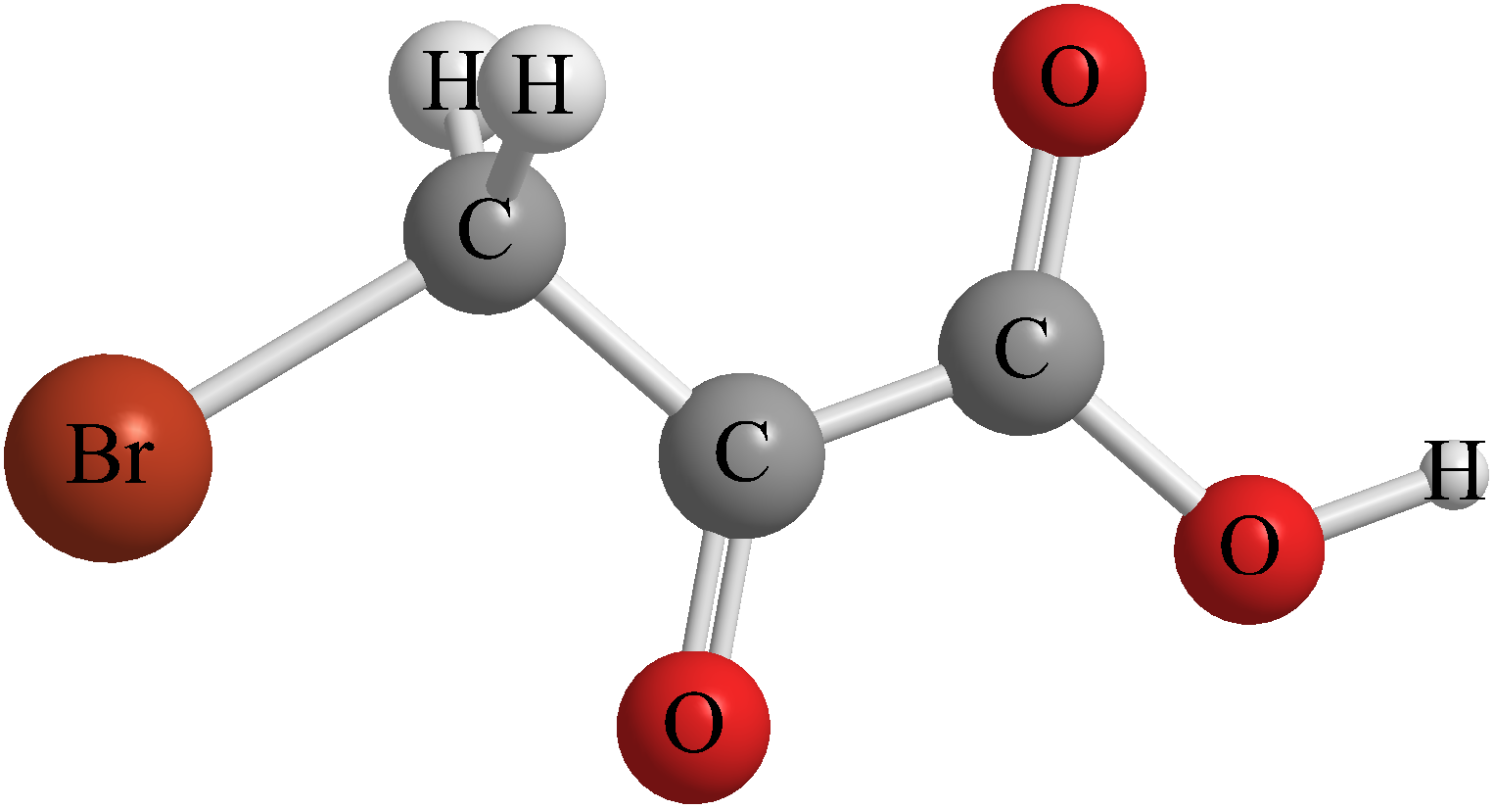
Chemical structure of 3-BrPA.

3-Bromopyruvate (3-BrPA; Fig. 1) is a hexokinase II (HK-II) inhibitor, showing potent inhibitory activity in the glycolysis process [9]. 3-BrPA demonstrates anticancer activity in a panel of cancer cell lines and animal tumor models [10]. Most of the known targets are thus involved in energy metabolism, and the anti-cancer effect of 3-BrPA is accordingly proposed to be due to the high dependence of tumor cells on glycolysis [26]. It is also reasonable to deduce that 3-BrPA can efficiently reverse the MDR of ABCB-1/P-gp overexpressing tumor cells, which with a high demand for ATP produced by glycolysis. Therefore, the objective of the present study is to characterize the biochemical changes caused by 3-BrPA using MCF-7/ADR cells in an attempt to elucidate the mechanisms underlying MDR reversal. The biochemical characterization was centered on the P-gp function and the ATP level. Our study should be beneficial to the ultimate elucidation of the mechanisms of MDR reversal by 3-BrPA.

## Materials and Methods

### Chemicals and reagents

3-BrPA, verapamil (VRP), paclitaxel, MTT and rhodamine 123(Rh123) were purchased from Sigma-Aldrich (Deisenhofen, Germany). Doxorubicin (Dox) and epirubicin (EPI) were purchased from Zhejiang HISUN Pharmaceuticals Co (Zhejiang, China). Daunorubicin was supplied by National Institute for the Food and Drug Control (Beijing, China). Mouse anti-ABCB-1/P-gp was obtained from Santa Cruz (CA, USA). Other antibodies were purchased from Cell Signaling Technology (Beverly, MA, USA). Alexa Fluor 488 goat anti-mouse IgG (H+L) was purchased from Life Technologies (Gaithersburg, MD, USA).

### Cell culture and cell viability

The breast cancer cell line MCF-7 and its drug-resistant variant MCF-7/ADR were kindly provided by Cancer institute & Hospital. Chinese Academy of Medical Sciences (Beijing, China).

These cell lines were maintained in RPMI1640 medium (Sigma, U.S.A.) supplemented with 10% fetal bovine serum (HyClone, U.S.A.), 100 units/mL penicillin G (Sigma, U.S.A.) and 100 µg/mL streptomycin (Sigma, U.S.A.). Cells were incubated in a humidified atmosphere with 5% CO_2_ at 37°C [12].

Cell viability was determined using the MTT assay. Cells were seeded in 96-well plates for 24h for cell attachment and were then incubated for 48 h with various concentrations (0.005–50 µM) of doxorubicin, paclitaxel, daunorubicin, and epirubicin in the presence or absence of 3-BrPA or VRP. MTT was then added into each well, and the cells were incubated for 4h [13]. Finally, the plates were centrifuged (1,000 rpm, 5 min) and the supernatant removed. The cell pellets were dissolved in 150 µL DMSO. The colored formazan products were quantified photometrically at 490 nm in a multi-mode microplate reader (Bio-Rad Laboratories, U.S.A.), and IC_50_ was then calculated using Graphpad Prism 5.0 [14].

### Animals and xenograft model

Female Balb/c nude, 4–6 weeks, 18–22 g weight were housed in a specific pathogen-free room using the MCF-7 and MCF-7/ADR xenografts. All experiment animals were purchased from the Guangdong Medical Laboratory Animal Center.

In our study, the models of xenografts of MCF-7 and MCF-7/ADR were established as follows: the breast cancer cell line MCF-7 and its drug-resistant variant MCF-7/ADR cells were collected and resuspended in PBS with an equal volume of Matrigel (BD,USA) and then were inoculated into the mice for chemotherapeutic studies. Mice received a subcutaneous (s.c.) injection of the cells under the armpit (10^7^ cells in 200µL). After s.c. implantation of the cells, the mice were randomized into four groups after the tumors reached approximately 100 mm^3^ in size, and then they received various regimens: control (normal saline, q2d×11, iv); 3-BrPA (q2d×11, iv, 5 mg/kg); EPI (q2d×11, iv, 0.5 mg/kg); 3-BrPA (q2d×11, iv, 5 mg/kg) and EPI (q2d×11, iv, 0.5 mg/kg) (3-BrPA and EPI were dissolved in normal saline and given 1 h before EPI was injected). The animals’ weight was measured every 2 days. We monitored tumor growth starting on the first day of treatment and measured the volume of the xenograft every 2 days. The tumor volume (V) was calculated using the formula V = π a*b^2^/6, where a and b are the longest and shortest diameters, respectively. Experiments were approved by the Laboratory Animal Ethics Committee Jinan University, China (Permit Number: 20131225001).

### Drug accumulation and efflux assay

For visualization of the effects of 3-BrPA on the intracellular retention of Rh123, 5×10^5^ cells were seeded on 6-well plate slides on the day prior to the assay and treatment with 3-BrPA (12.5, 25 µM) or 10 µM VRP for 4 h at 37°C. Then cells were incubated with either 5 µM Rh123 alone or a 5 µM Rh123 complex with 3-BrPA or VRP in fresh RPMI1640 medium for 1 h in darkness at 37°C. After the cells were washed for three times with cold PBS, images were acquired by fluorescence microscopy (Olympus, Japan) 488 nm excitation and 535 nm emission [15] wavelength [16].

To make a further quantitative analysis of Rh123 retention, 1×10^4^ cells/well were seeded in 96-well black plates, cultured overnight, and treated with 3-BrPA (12.5, 25 µM) or 10 µM VRP for 4 h at 37°C. Cells were then incubated with either 5 µM Rh123 alone or in the presence of 3-BrPA or VRP in fresh RPMI1640 medium for 1 h at 37°C. After washing cells for three times with cold PBS, the fluorescent intensity was detected by multi-mode microplate reader at 488 nm excitation and 535 nm emission wavelengths.

Simultaneously, Rh123 efflux was also quantified in this study, 1×10^4^ cells/well were seeded in 96-well black plates and cultured overnight. Cells were treated with 3-BrPA (12.5, 25 µM) or 10 µM VRP for 4 h at 37°C. Then 5 µM Rh123 or 5 µM Rh123 with 3-BrPA as well as VRP was cultured in fresh RPMI1640 medium for 1 h at 37°C. After the removal of Rh123, an exclusion step was performed in the presence or absence of 3-BrPA or VRP, and then the cells were washed three times with cold PBS at the desired time (0, 30, 60, 90, or 120 min) and measured spectrofluorometrically at 488 nm excitation and 535 nm emission wavelength using a multi-mode microplate reader [17].

The intracellular amount of EPI was quantitatively determined by flow cytometry (Beckman Coulter, U.S.A.). One milliliter of cell suspension (1×10^6^ cells/mL) was treated with various concentrations of 3-BrPA (12.5, 25 µM) or 10 µM verapamil for 4 h at 37°C and then incubated with 10 µM EPI for an additional 1 h in darkness at 37°C, respectively. After that, cells were collected by centrifugation at 1,000 rpm and washed for three times with cold PBS. Finally, the cells were re-suspended in PBS buffer and analyzed by flow cytometry (Beckman Coulter, U.S.A.) equipped at an excitation wavelength of 488 nm and an emission wavelength 590 nm, respectively [18].

### Intracellular ATP measurement

Intracellular ATP levels were measured using a firefly luciferase based ATP Assay Kit (Beyotime, China) according to the manufacturer’s instructions. In brief, 5×10^4^ cells/well were seeded in 12-well plates for 24 h to allow for cell attachment and were then incubated with or without 3-BrPA (12.5, 25 µM) in RPMI1640 medium for 48 h at 37°C. After every well plate, 100 µL of each supernatant was mixed with 100 µL ATP detection working dilution. Luminance (RLU) was measured by multi-mode microplate reader. The protein of each treatment group was determined by the BCA Protein Assay Kit (Beyotime, China). The total ATP levels were expressed as n mol/mg protein [19].

### Hexokinase-II bioactivity

The activity of Hexokinase-II (HK-II) was examined by HK Assay Kit (Nanjinjiancheng, China). In brief, cells were seeded in plates cultured overnight to allow for cell attachment and were then incubated with or without 3-BrPA (12.5, 25 µM) in RPMI1640 medium for 48 h at 37°C. After that, the activities of HK-II bioactivity were detected by ultraviolet spectrophotometry (Thermo, USA) [20].

### Real-time PCR detection of mRNA

The expression of a cancer resistance protein gene was analyzed at the mRNA and the protein level in this study. The mRNA expression was determined by real-time PCR. Total RNA was isolated from cells and tumor tissues using the Trizol reagent (Takara, Japan) according to the manufacturer’s instructions [21]. Complementary DNA (cDNA) corresponding to 0.8 µg of total RNA was used per reaction (20 µL) in a real-time quantitative PCR reaction performed on a Roche Light Cycler (Mannheim, Germany) and using Power SYBER Green Master Mix (Takara, Japan). The primers as shown in Table 1.

**Table 1:** Primers used for real-time PCR.

Gene	Forward primer 5′-3′	Reverse primer 5′-3′
ABCA-1	CAATCTCACCACTTCGGTCTCCA	CTCTTCTCATCACTTTCCTCGCC
ABCB-1[29]	TGGTGTTTGGAGAAATGACAGATAT	ACCAATTCCACTGTAATAATAGGCA
ABCC-1	AAATAGAGACTGAGAGTGAGCAACC	CATGAGAGGGAAAGAAAAGAGG
ABCC-2	CCTATGTCCCACAGCAGTCCT	ATTTATACCCTTCTCTCCAATCTCA
ABCC-3	CTGTTTTCTTTGTCACCCCCTTG	CAGAAGATAATGAGGACCCCCG
ABCG-1	AAGGGGGTCGCTCCATCATTT	GGTTGTGGTAGGTTGGGCAGTT
ABCG-2	AAAGGAACCCAAGGAGATAGGAG	GCAGGAGAAAGAATGAGAGAGGAAA
β-actin [30]	GTTGCGTTACACCCTTTCTTGAC	CTCGGCCACATTGTGAACTTTG

### Western blotting

Protein lysates collected from cells and tumor tissues were resolved by SDS-PAGE and transferred onto PVDF membranes (0.22 µm). The membranes were incubated with the desired primary antibody for P-gp (1∶1,000) and β-actin (1∶1,000) overnight at 4°C, followed by incubation with the appropriate secondary antibody for 2 h at room temperature. The detection of β-actin was used as a loading control [22].

### ATPase activity

The ATPase activity was determined by P-gp-Glo Assay Systems with P-glycoprotein (Promega, U.S.A.). Sodium vanadate (Na_3_VO_4_) was used as a P-gp ATPase inhibitor. The activity of P-gp ATPase was measured in the presence of 3-BrPA, in accordance with the instructions. The sample luminescence reflects the ATP level, which is negatively correlated with the activity of P-gp ATPase and recorded using the multi-mode microplate readers [17]. The activity detected in cells treated with the test compound is expressed as the percentage of basal activity. By comparing the basal activity to the activity after exposure to test compounds, the compounds can be ranked as stimulating, inhibiting, or having no effect on basal P-gp ATPase activity.

### Data analysis

Each experiment was conducted at least three times, and the data are expressed as mean ± SD. A significant difference was determined using one-way ANOVA analysis. A value of *P*<0.05, *P*<0.01, or *P*<0.001 was considered statistically significant.

The multidrug resistance ratio (MR) was defined to evaluate the extent of cell resistance to the anti-cancer drugs:
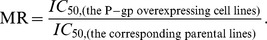



The reversal ratio (RR) was defined to evaluate the ability of a reversing agent to reverse the multidrug resistance:
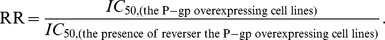



## Results

### MDR characterization in MCF-7/ADR cells

We determined the IC_50_ values of several anti-cancer drugs in both MCF-7/ADR cells and their parental cells (Table 2). The multidrug resistance ratios (MRs) were 23, 82, 288, and 121 for doxorubicin, paclitaxel, daunorubicin, and epirubicin, respectively. The results indicated that MCF-7/ADR was a suitable cell line for the evaluation of the MDR reversal capability of 3-BrPA.

**Table 2:** Effect of 3-BrPA on reversing ABCB-1/P-gp mediated drug resistance in MCF-7 and MCF-7/ADR.

Drug	Combined medication		IC_50_± SD^a^ (µM) (fold-reversal)^b^	
			MCF-7	MCF-7/ADR
doxorubicin	3-BrPA (µM)	0	0.41±0.021(1.00)	9.35±0.039(1.00)
		12.5	0.62±0.004(0.66)	0.46±0.032(20.33)**
		25	0.40±0.054(1.02)	0.03±0.001(283.33)** ^,^ #
	VRP (µM)	10	0.38±0.017(1.08)	0.45±0.040(20.92)**
Paclitaxel	3-BrPA (µM)	0	0.05±0.001(1.00)	4.08±0.032(1.00)
		12.5	0.047±0.007(1.06)	0.477±0.095(8.55)**
		25	0.046±0.005(1.09)	0.048±0.007(85)** ^,^ #
	VRP (µM)	10	0.045±0.001(1.01)	0.463±0.015(8.81)**
daunorubicin	3-BrPA (µM)	0	0.078±0.003(1.00)	22.47±0.033(1.00)
		12.5	0.077±0.003(1.01)	0.112±0.003(200.6)**
		25	0.078±0.008(1.00)	0.105±0.004(214)**
	VRP (µM)	10	0.079±0.009(0.99)	0.112±0.001(200.6)**
epirubicin	3-BrPA (µM)	0	0.153±0.008(1.00)	18.49±0.097(1.00)
		12.5	0.245±0.071(0.62)	0.122±0.042(151.6)**
		25	0.213±0.036(0.72)	0.108±0.003(171.2)**
	VRP (µM)	10	0.105±0.018(1.46)	0.196±0.001(96.4)**

Cell survival was determined by MTT assay as described in Materials and Methods.

a Data in the table are shown as the means ± SD (n = 6) of at least three independent experiments.

b RR value indicating the fold reversal of MDR.

**P<0.01,*P<0.05 for the IC_50_ versus that absence of 3-BrPA.

# *P*<0.05 for the IC_50_ versus that VRP.

### ABCB-1/P-gp overexpression in MCF-7/ADR cells

**Figure 2:**
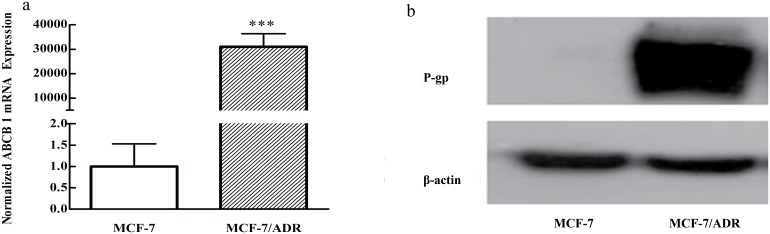
P-gp overexpression in MCF-7/ADR cell lines. a: ABCB-1 expression level in MCF-7 vs. MCF-7/ADR cells by real-time PCR b: P-gp expression level in MCF-7 vs. MCF-7/ADR cells by Western blotting ***P<0.001 versus the parental cells.

The mRNA level of ABCB-1 in MCF-7/ADR cells was significantly higher (about 30,000 times) than that in MCF-7 (Fig. 2a). In addition, the P-gp protein was overexpressed in the multidrug resistance cell line, whereas it was hardly found in the parental cells (Fig. 2b).

### MDR reversal by 3-BrPA in MCF-7/ADR cells

**Figure 3:**
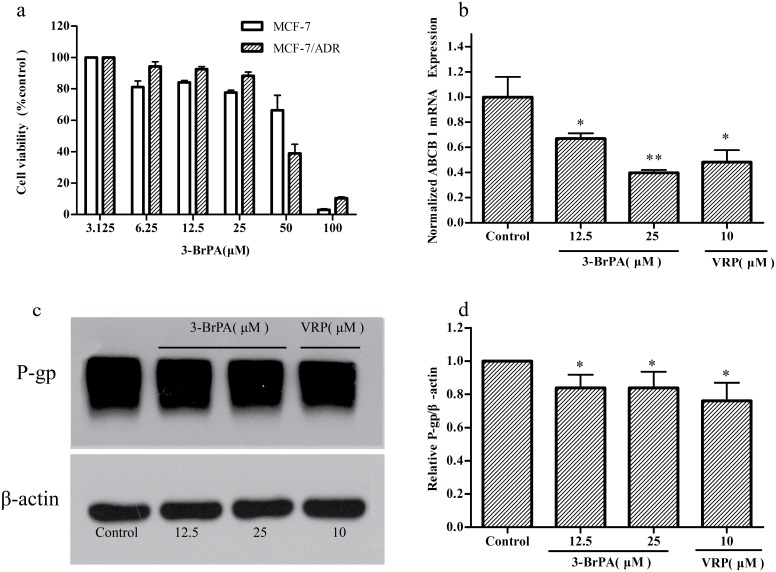
The cytotoxicity of 3-BrPA, effect of 3-BrPA on the mRNA and protein expression levels of ABCB-1/P-gp. These cell lines were exposed to the indicated concentration of 3-BrPA for 48 h, and cell viability was determined by MTT assay. Each points represents the mean ± SD (n = 6). Each experiment was performed three times. a: The cytotoxicity of 3-BrPA in MCF-7, MCF-7/ADR,After treated with desired concentrations of 3-BrPA for 48 h b: The mRNA level of P-gp in MCF-7/ADR cells treated with or without 3-BrPA was determined by real –time PCR, β-actin served as internal controls for real-time PCR respectively. c, d: The protein expression level of P-gp in MCF-7/ADR cells treated with or without 3-BrPA was determined by Western blotting analysis, β-actin served as internal controls Western blotting, respectively. *P<0.05 compared with the control, **P<0.01compared with the control.

The cytotoxicity of 3-BrPA was evaluated using MTT assay. 3-BrPA showed significant cytotoxicity when the dose was 50 µM or higher (Fig. 3a). Therefore, two dosing levels (12.5 µM and 25 µM) that did not show obvious cytotoxicity were chosen to assess their MDR reversal ability. 3-BrPA significantly decreased the IC_50_ values of doxorubicin, paclitaxel, daunorubicin, and epirubicin in MCF-7/ADR cells, whereas it did not alter the IC_50_ values in MCF-7 cells (Table 2). The reversal ratios (RRs) were 20–283, 9–85, 201–214 and 152–171 for doxorubicin, paclitaxel, daunorubicin, and epirubicin, respectively. The results indicated that 3-BrPA significantly reversed the resistance of the cells to the typical chemotherapeutics that are P-gp substrates [23].

### The changes of ABCB-1/P-gp expression caused by 3-BrPA *in vitro*

The use of 3-BrPA led to slight decreases (of 0.3–0.6 times) in the mRNA level of ABCB-1 in MCF-7/ADR cells (Fig. 3b). Meanwhile, the expression of P-gp protein was also slightly reduced by 0.17 and 0.2 in the presence of 3-BrPA (12.5, 25 µM) (Fig. 3c and 3d).

### 3-BrPA enhancement of the uptake of Rh123 and EPI in MCF-7/ADR cells

**Figure 4:**
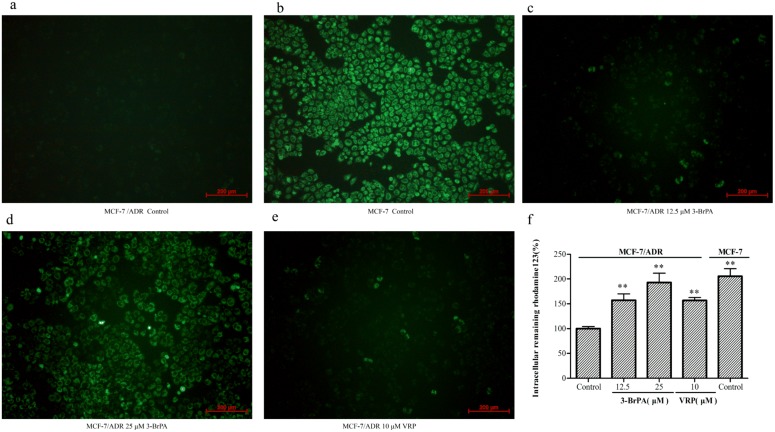
3-BrPA and VRP increase the accumulation of Rh123 in MCF-7/ADR cells. Cells were treated with 12.5, 25 µM 3-BrPA and 10 µµ VRP for 4 h at 37°C. Then Rh123 (5 µM) was added and incubated in the dark at 37°C for an additional 1 h. After washing cells three times with cold PBS, images were acquired by a fluorescence microscopy at 488 nm extraction and 535 nm emission wavelength for Rh123. The fluorescence intensity was quantitatively detected by multi-mode microplate reader. a-e: 3-BrPA and VRP increase the accumulation of Rh123 in MCF-7/ADR by fluorescence microscopy f: 3-BrPA and VRP increase the accumulation of Rh123 in MCF-7/ADR by multi-mode microplate reader **P<0.05 compared with the control.

**Figure 5:**
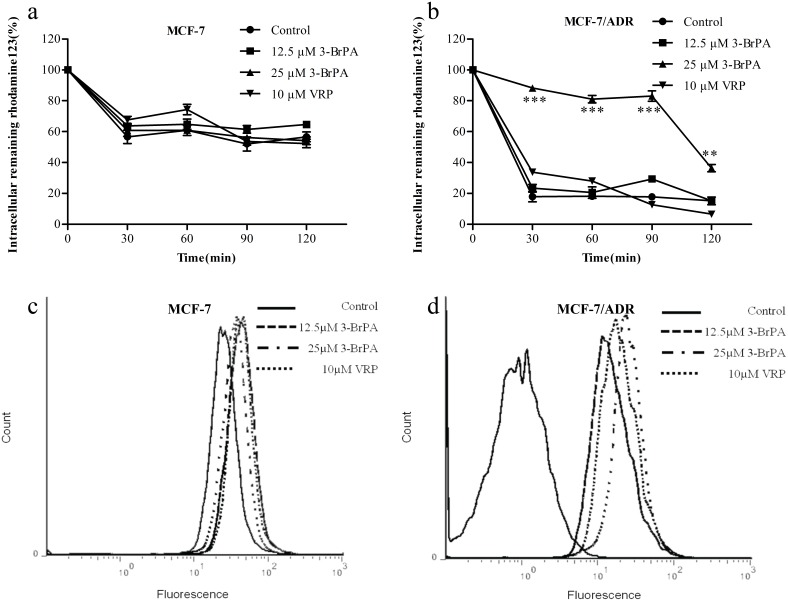
Inhibition of Rh123 efflux from MCF-7/ADR cells by 3-BrPA and VRP. 3-BrPA and VRP increase the accumulation of EPI in cells. a,b: After incubation 1 h in the presence of Rh123 (5 µM) and 3-BrPA (12.5, 25 µM) and VRP (10 µM),the cells were washed and incubated in fresh medium for indicated times. Each point represents the mean ± SD (n = 6). Each experiment was performed three times. c,d: These cells were incubated with 3-BrPA (12.5, 25 µM) at 37°C for 4 h, then 10 µM EPI was added for another 1 h incubation. Intracellular fluorescence was analyzed by flow cytometry. Control cells that were not exposed to any 3-BrPA, and VRP (10 µM) were used as positive control. The change of intracellular fluorescence in MCF-7 and MCF-7/ADR ***P<0.01 compared with the control.

The accumulation of Rh123 was greatly enhanced by 3-BrPA in the resistant cells, evidenced by the increased fluorescent intensity in the presence of the compound (Fig. 4a). The extent of enhancement was 1.8 times, as revealed by the quantitative analysis (Fig. 4b). To be specific, the percentage of remaining intracellular Rh123 at 30, 60, 90, and 120 min in MCF-7/ADR is 88%, 83%, 81%, and 37% of control, respectively, after incubation with 25 µM 3-BrPA (Fig. 5b). These results suggested that 3-BrPA can modify the transport property of Rh123, which may result from the inhibition of ABCB-1/P-gp functioning.

The EPI uptake in MCF-7/ADR cells was measured using flow cytometric analysis [24]. The results showed that the location of the fluorescence peak gradually shifted to the right (about 90%) in the presence of 3-BrPA in relation to its absence (Fig. 5c). However, the changes in EPI uptake were negligible in MCF-7.

### 3-BrPA decreased the intracellular level of ATP and inhibited the HK-II bioactivity in MCF-7/ADR cells

**Figure 6:**
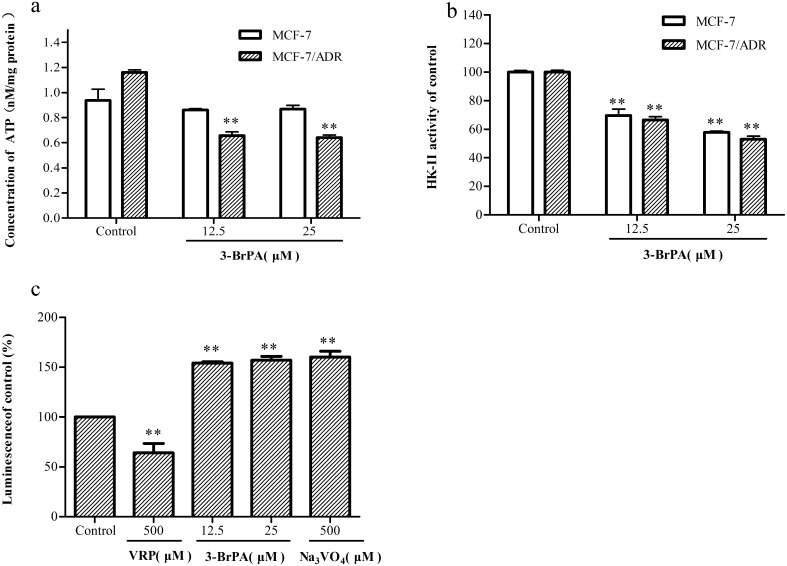
Reduction in ATP production and inhibition of HK-II and ABCB-1 ATPase activity of cells by 3-BrPA. After the treatment with 3-BrPA (12.5, 25 µM), the ATP levels and HK-II activity were determined by ATP Assay Kit and HK Assay Kit respectively. Each point represents the mean ± SD (n = 6). The ATPase activity of ABCB-1 was determined in recombinant human ABCB-1 membranes treated with 3-BrPA compared with Na_3_VO_4_. Each point represents the mean ± SD (n = 6) for at least three replicates. a: The effect of 3-BrPA on ATP level in MCF-7 and MCF-7/ADR b: The effect of 3-BrPA on HK-II activity in MCF-7 and MCF-7/ADR c: The effect of 3-BrPA on ABCB-1 ATPase activity in MCF-7 and MCF-7/ADR **P<0.01 compared with the control.

The ATP levels, which were only 0.13 and 0.14 n mol/mg protein in the parental cells, were significantly decreased inside of the resistant cells by 0.51 and 0.52 n mol/mg protein by 3-BrPA (Fig. 6a). Furthermore, 3-BrPA (12.5–25 µM) inhibited almost half of the HK-II activity in both resistant and parental cells (Fig. 6b).

The biological actions of 3-BrPA appear to be rather complex [25], [26]. The important intracellular proteins that interacted with 3-BrPA included HK-II [27], GAPDH, and mitochondrial succinate dehydrogenase. The decrease in the ATP level was most likely resulted from HK-II inhibition associated with the reduced ATP production from glycolysis [28]. This is supported by the fact that the HK-II activity was inhibited by 3-BrPA in the resistant cells (Fig. 6b).

### 3-BrPA showed inhibitory effects on ATPase activity

The inhibitory effects of 3-BrPA on ATPase activity was assessed using the ATPase Assay Kit. 3-BrPA showed strong inhibition on ATPase activity, and the inhibitory potency of 3-BrPA (12.5 and 25 µM) was comparable to that of sodium vanadate (Fig. 6c).

### Reversal of MDR by 3-BrPA in MCF-7/ADR xenografts

**Figure 7:**
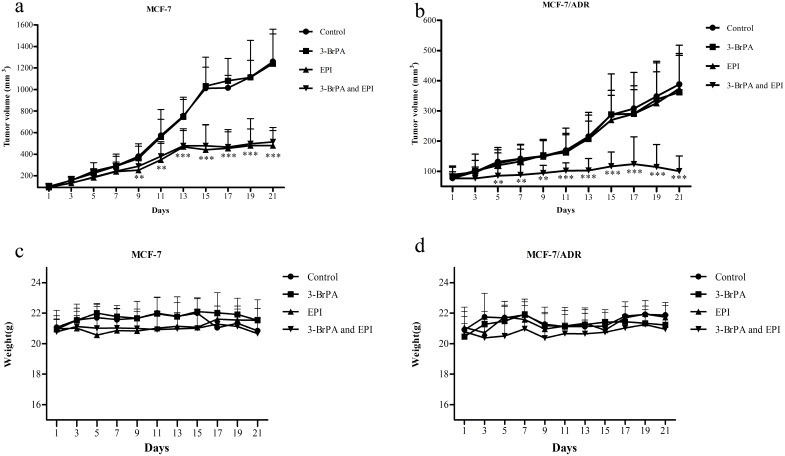
The MDR reversal effect of 3-BrPA on MCF/ADR xenograft model. The treatments were as follows: control (normal saline, q2d×11, iv); 3-BrPA (q2d×11, iv, 5 mg/kg); EPI (q2d×11, iv, 0.5 mg/kg); 3-BrPA (q2d×11, iv, 5 mg/kg) and EPI (q2d×11, iv, 0.5 mg/kg), 3-BrPA and EPI were dissolved in normal saline, and was given 1 h before EPI was injected, mean of tumor volume for each group (n = 6) after implantation. Each point on line graph represents the mean of tumor volume (mm^3^) at a particular day after implantation, and each bar represents SD. Potentiation of antitumor effects of EPI by 3-BrPA in ABCB-1/P-gp overexpressing MCF-7/ADR xenograft model is shown. a: Changes in the mean of tumor volume over the time course of the experiment in MCF-7 xenograft model. b: Changes in tumor volume over the time course of the experiment in ABCB-1/P-gp overexpressing MCF-7/ADR xenograft model are shown. c: Changes in the mean of body weight over the time course of the experiment in MCF-7 xenograft model are shown. d: Changes in the mean of body weight over the time course of the experiment in ABCB-1/P-gp overexpressing MCF-7/ADR xenograft model are shown. ***P<0.001 versus the control group; **P<0.01 versus the control group.

The MCF-7/ADR cells were exposed to 25 µM 3-BrPA, achieving the maximum potentiation of EPI cytotoxicity (171-fold, Table 2). 3-BrPA was also found to potentiate significantly the antitumor activity of EPI against MCF-7/ADR xenografts *in vivo* (Fig. 7).

No significant difference was observed in tumor size between experimental animals treated with saline and EPI in MCF-7/ADR. However, the mean tumor size in the EPI group was significantly smaller than that of the saline groups in MCF-7 (Fig. 7a and 7b). The results indicated that it is a suitable *in vivo* model for evaluation of the MDR reversal capability of 3-BrPA.

**Figure 8:**
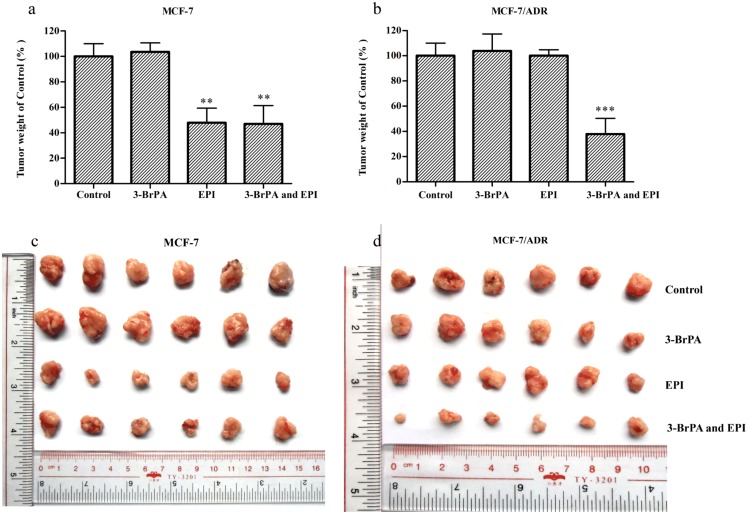
The changes of tumor weight by 3-BrPA on MCF/ADR xenograft model. a: The bar graph represents the mean of tumor weights (mice, n = 6) of the excised MCF-7 tumor from different mice. b: The bar graph represents the mean of tumor weights (mice, n = 6) of the excised ABCB-1/P-gp overexpressing MCF-7/ADR tumor from different groups. c:A representative picture of the excised MCF-7 tumor sizes from different groups is shown on the 21th day after implantation. d: A representative picture of the excised ABCB-1/P-gp overexpressing MCF-7/ADR tumor sizes from different groups is shown on the 21th day after implantation. Each group represents the mean of determinations, and the bar represents SD. ***P<0.001 versus the control group; **P<0.01 versus the control group.

Treatment of 3-BrPA tumor-bearing nude mice with EPI (0.5 mg/kg, i.v.) or 3-BrPA (5 mg/kg, i.v.) alone had little or no effect on the growth rate of the tumors (Fig. 7b and Fig. 8). However, 3-BrPA plus EPI reduced the growth rate of the tumors significantly (Fig. 7b and Fig. 8). Remarkably, the regimen of the combination of EPI and 3-BrPA did not cause any deaths in the experimental process. There was no substantial or reproducible increase in body weight loss in animals treated with EPI plus 3-BrPA compared with the drug-alone groups (Fig. 7c and 7d). This suggests that this regimen did not result in increased toxic side effects. Therefore, the toxicity of co-administration of EPI and 3-BrPA was tolerable.

### Down-regulation of ABCB-1/P-gp expression caused by 3-BrPA *in vivo*

**Figure 9:**
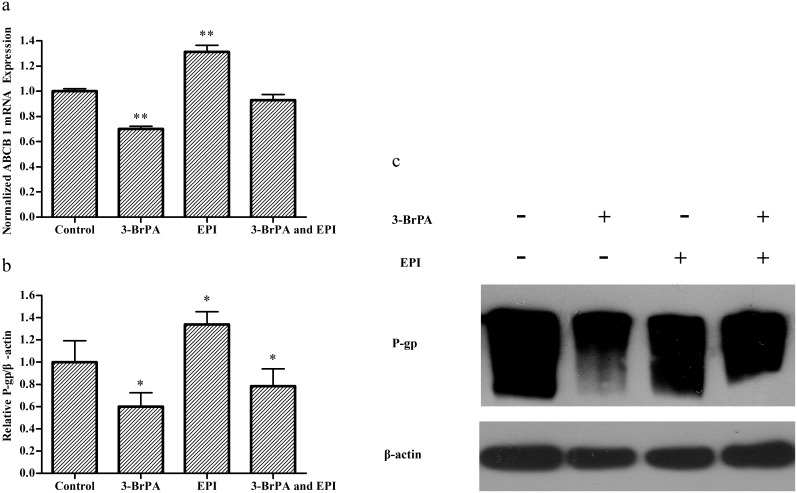
ABCB-1/P-gp expression changes caused by 3-BrPA *in vivo*. a: The mRNA level of ABCB-1 in MCF-7/ADR tumor tissue treated with different groups was determined by real-time PCR, β-actin served as internal controls for real-time PCR respectively. b, c: The protein expression level of P-gp in MCF-7/ADR tumor tissue treated with different groups was determined by Western blotting analysis, β-actin served as internal controls Western blotting, respectively. *P<0.05 compared with the control, **P<0.01 compared with the control.

To investigate the significance of ABCB-1/P-gp down-regulation *in vivo*, we assessed ABCB-1/P-gp expression in tumor tissues. Real-time PCR and Western blotting results demonstrated that treatment with 3-BrPA resulted in 30% ABCB-1 mRNA and 40% P-gp protein reduction of normal saline in MCF-7/ADR xenografts, respectively (Fig. 9a and 9b). Interestingly, the increased ABCB-1 mRNA and P-gp protein levels in the EPI group’s tumor tissue were confirmed by real-time PCR and Western blotting. However, the ABCB-1/P-gp expression of the 3-BrPA plus EPI group was significantly smaller than that of the EPI group in MCF-7/ADR tumor tissue. These data indicate that 3-BrPA inhibits the expression of ABCB-1/P-gp *in vivo*.

## Discussion

A broad range of MDR reversal modulators that interact with ABC transporters have been reported in preclinical or clinical trials since the advent of the first-generation MDR reversal agents (e.g., VRP and cyclosporine A). However, there are two major defects as that enhancing the toxicity of the cytotoxic drugs and relative non-specificity with weak effect. All of these obstacles clearly indicate that the development of potential MDR reversal modulators with low toxicity is an important approach to the research on modulators. Recently, Ayako Nakano found that glycolysis inhibition restores the susceptibility of ABC transporter-expressing cells to chemotherapeutic agents [11]. 3-BrPA is a potent inhibitor of HK-II and effectively inhibits glycolysis. Most of the known targets are thus involved in energy metabolism, and the anti-cancer effect of 3-BrPA is accordingly proposed to be due to the high dependence of tumor cells on glycolysis [26]. It is also reasonable to deduce that 3-BrPA can efficiently reverse the MDR of ABCB-1/P-gp overexpressing tumor cells, which with a high demand for ATP produced by glycolysis.

The present *in vitro* and *in vivo* studies demonstrated that 3-BrPA is a more potent modulator of P-gp mediated MDR than the first-generation positive modulator VRP. The *in vitro* and *in vivo* potency were evaluated by several assays using a drug-resistant variant MCF-7/ADR with a high level of ABCB-1/P-gp special expression (*Fig. S1*), which was confirmed by real-time PCR in our studies. MTT assay was used to assess quantitatively the effect of 3-BrPA by calculating the IC_50_ and the reversal ratios (RRs) after the post addition of cytotoxic drugs. To preclude the intrinsic cytotoxicity of 3-BrPA, a non-cytotoxic concentration of 3-BrPA was selected to be used in our studies, and the results showed that 3-BrPA was very potent in reversing the resistance of MCF-7/ADR cell lines. The same assay was also used for MCF-7 to determine whether 3-BrPA could function with the cytotoxic drugs to reverse MDR or not. As the results showed, 3-BrPA did not influence the IC_50_ of the wild-type MCF-7 cells, confirming the combined sensitizing effect of 3-BrPA. Based on the above results, further experiments were conducted *in vivo*. The ability of 3-BrPA to reverse P-gp-mediated MDR *in vivo* was evaluated using MCF-7 and MCF-7/ADR xenograft models in combination with cytotoxic drug EPI to investigate the effect of 3-BrPA *in vivo*. As the data showed, EPI alone significantly reduced the growth rate of the parental sensitive MCF-7 cell line xenograft and that the co-administration of 3-BrPA did not enhance the activity of EPI. In contrast, EPI used alone had no effect on the growth rate of the MCF-7/ADR resistant cell line tumors, while the co-administration of 3-BrPA restored the antitumor activity of cytotoxic agent. 3-BrPA showed great promise in reversing cancer cell resistance to chemotherapeutics in the present data.

Given the results shown above, we investigated whether 3-BrPA could inhibit the efflux of ABCB-1 to enhance the cytotoxicity of the agents by increasing the intracellular drug concentration as following two parts. We first observed the accumulation Rh123 a substrate of ABCB-1 by spectrofluorometrically, multi-mode microplate reader, and flow cytometry, suggesting that the intracellular accumulation of Rh123 and EPI was enhanced under the presence of 3-BrPA. However, we also found that the degree of the fluorescence decay of Rh123 in the presence of 3-BrPA was significantly slower than in the control group, which showed that the effect of 3-BrPA on enhancing the accumulation of Rh123 in resistant cells was closely related to its power in inhibiting the efflux of Rh123. As much evidence has shown, the resistant cell lines frequently ABCB-1/P-gp overexpress cells, showing a positive correlation with the degree of resistance of the cells.

Many compounds show the power to reverse resistance in tumor cells by down the expression of ABCB-1/P-gp. In our previous studies, the real-time PCR and Western blotting results showed that ABCB-1/P-gp did drop slightly at the mRNA and protein levels in MCF-7/ADR cells and tumor tissue. More interestingly, this is the first time we have found that 3-BrPA could influence the distribution of P-gp (*Fig. S2*). We speculate that 3-BrPA ubiquitin ligases affect protein turnover. What is worth more attention is that 3-BrPA could reverse the resistance of tumor cells by depleting ATP and inhibiting the activity of HK-II at the same time.

One of the major reason to inhibit the activity of ABCB-1/P-gp activity is to deplete ATP. Thus, we investigated the effect of 3-BrPA on the function of ABCB-1/P-gp. ABCB-1 is a transporter protein with two trans-membrane domains (TMD) and two nucleotide binding domains (NBD). A substrate can get into the substrate-binding region favorably when it enters the cell through the phospholipid bilayer, which will stimulate the release of ATP. ATP hydrolysis plays an important role in this process, so ATPase plays a key role in influencing the ABCB-1 function on transporting drugs. The data from our studies indicated that 3-BrPA had a direct inhibitory effect on ATPase activity, which also illustrated that 3-BrPA could influence ABCB-1 function by inhibiting the ATPase.

3-BrPA was shown to be a great modulator of P-gp-mediated MDR *in vitro* and *in vivo* with glorious potential. *In vivo*, 3-BrPA appeared to be well tolerated, whether used alone or in combination with cytotoxic agents, as indicated by the minimal change in body weight and the zero mortality rates. Meanwhile, the HE assay also showed that there was no apparent tissue necrosis in all major organs (*data not shown*). However, in our research on 3-BrPA, we also found that the most prominent feature of 3-BrPA was the low stability. Therefore, further research will be conducted on 3-BrPA to reform its structure, trying to obtain a stable, targeted MDR reversal agent with low-toxicity. To the best of our knowledge, the great reversal activity of 3-BrPA may remind us not only that the macromolecule compounds have great reversal activity but also that a small molecule is likely to be achieved easily with high efficiency and sometimes low toxicity. Finally, it is expected that all the workon 3-BrPA will provide useful clues for further research on MDR reversal agents. Elucidation of the MDR reversal mechanism is of interest to pharmacologists, as the information would provide guidance on whether it is possible to develop 3-BrPA as an adjuvant MDR-reversing agent in chemotherapy.

## Supporting Information

Figure S1 **ABC family genes expression** ***in vitro*** **and** ***in vivo.*** a: The mRNA level of ABCA-1,ABCB-1, ABCC-1, ABCC-2, ABCC-3,ABCG-1,ABCG-2 in MCF-7 and MCF-7/ADR cells. b: The mRNA level of ABCA-1,ABCB-1, ABCC-1, ABCC-2, ABCC-3,ABCG-1,ABCG-2 in MCF-7 and MCF-7/ADR tumor tissues.
(TIF)

Figure S2 **3-BrPA could influence the distribution of P-gp.**
(TIF)
