# Supplementary figures and images for: The Reversal Effects of 3-Bromopyruvate on Multidrug Resistance In Vitro and In Vivo Derived from Human Breast MCF-7/ADR Cells
Source: PLoS One. 2014 Nov 5;9(11):e112132. doi: 10.1371/journal.pone.0112132 (PMC4221289; doi:10.1371/journal.pone.0112132)

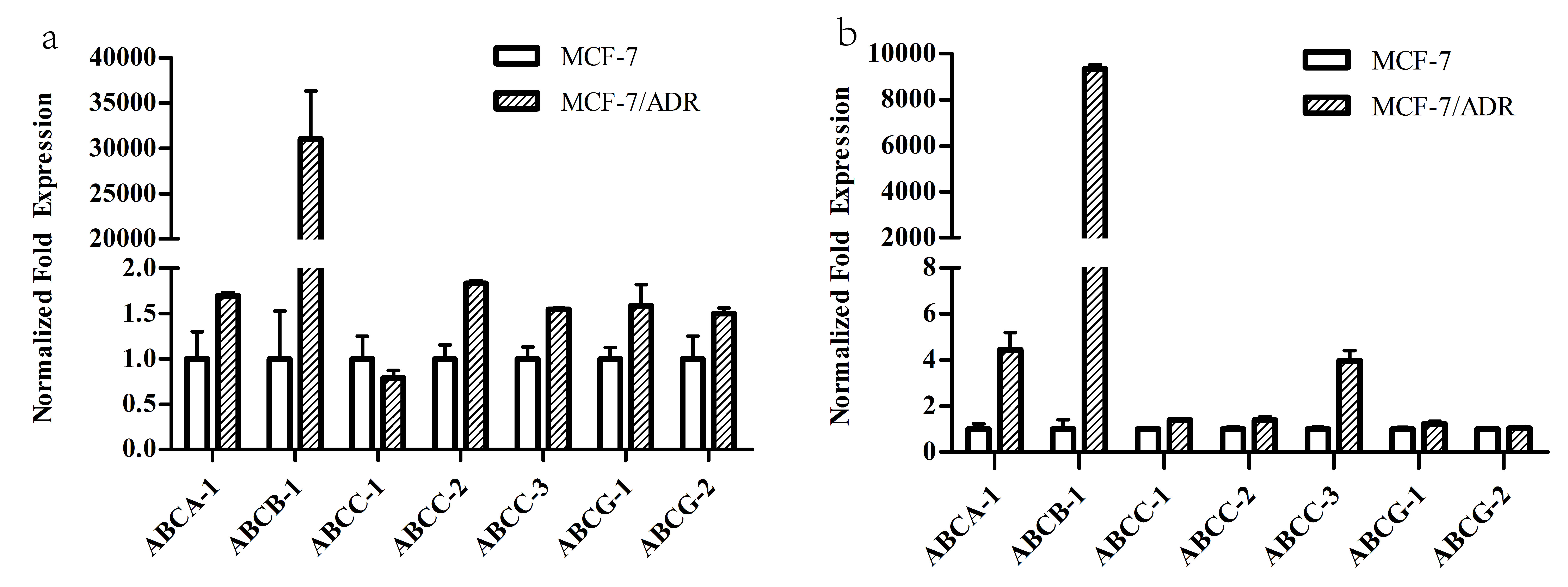

Supplement: Figure S1 — ABC family genes expression in vitro and in vivo. a: The mRNA level of ABCA-1,ABCB-1, ABCC-1, ABCC-2, ABCC-3,ABCG-1,ABCG-2 in MCF-7 and MCF-7/ADR cells. b: The mRNA level of ABCA-1,ABCB-1, ABCC-1, ABCC-2, ABCC-3,ABCG-1,ABCG-2 in MCF-7 and MCF-7/ADR tumor tissues. (TIF) [file pone.0112132.s001.tif]

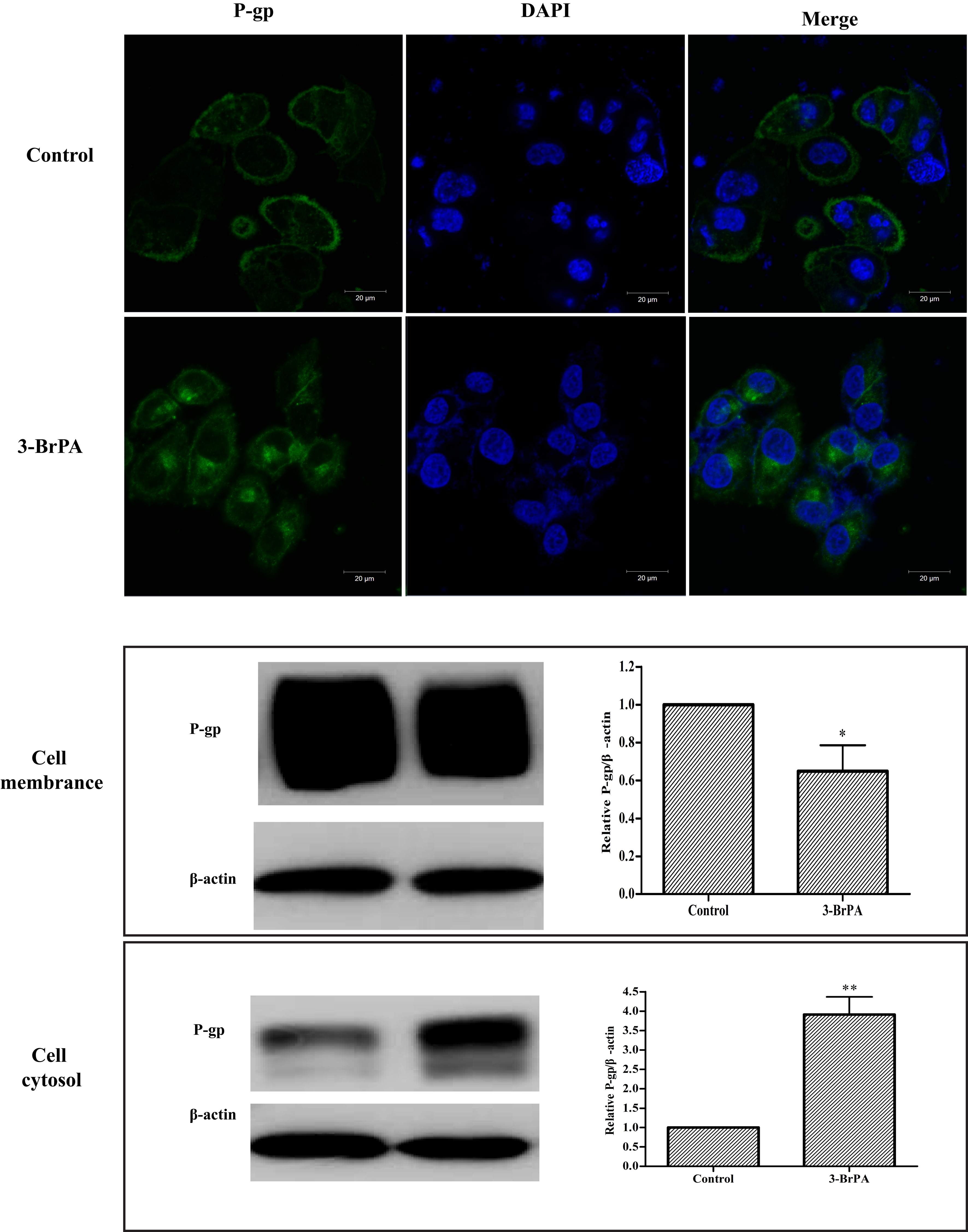

Supplement: Figure S2 — 3-BrPA could influence the distribution of P-gp. (TIF) [file pone.0112132.s002.tif]
